# Evaluation of Oxidative Stress and Mitophagy during Adipogenic Differentiation of Adipose-Derived Stem Cells Isolated from Equine Metabolic Syndrome (EMS) Horses

**DOI:** 10.1155/2018/5340756

**Published:** 2018-06-06

**Authors:** Krzysztof Marycz, Christine Weiss, Agnieszka Śmieszek, Katarzyna Kornicka

**Affiliations:** ^1^Department of Experimental, The Faculty of Biology and Animal Science, Wroclaw University of Environmental and Life Sciences, Norwida 25, 50-375 Wroclaw, Poland; ^2^Wroclaw Research Centre EIT+, Stablowicka 147, 54-066 Wroclaw, Poland; ^3^PferdePraxis Dr. Med. Vet. Daniel Weiss, Postmatte 14, CH-8807 Freienbach, Switzerland

## Abstract

Mesenchymal stem cells (MSCs) are frequently used in both human and veterinary medicine because their unique properties, such as modulating the immune response and differentiating into multiple lineages, make them a valuable tool in cell-based therapies. However, many studies have indicated the age-, lifestyle-, and disease-related deterioration of MSC regenerative characteristics. However, it still needs to be elucidated how the patient's health status affects the effectiveness of MSC differentiation. In the present study, we isolated mesenchymal stem cells from adipose tissue (adipose-derived mesenchymal stem cells (ASCs)) from horses diagnosed with equine metabolic syndrome (EMS), a common metabolic disorder characterized by pathological obesity and insulin resistance. We investigated the metabolic status of isolated cells during adipogenic differentiation using multiple research methods, such as flow cytometry, PCR, immunofluorescence, or transmission and confocal microscopy. The results indicated the impaired differentiation potential of ASC_EMS._ Excessive ROS accumulation and ER stress are most likely the major factors limiting the multipotency of these cells. However, we observed autophagic flux during differentiation as a protective mechanism that allows cells to maintain homeostasis and remove dysfunctional mitochondria.

## 1. Introduction

Mesenchymal stem cells (MSCs) can be isolated from multiple sources, including bone marrow and adipose tissue [1, 2]. MSCs from adipose tissue (adipose-derived mesenchymal stem cells (ASCs)) can be easily obtained with a minimal invasive procedure, thus much attention has been paid to their clinical application [3–5]. ASCs are multipotent cells capable of differentiating into osteogenic, chondrogenic, and adipogenic lineages. They are characterized by the presence of specific surface antigens, including CD73, CD90, and CD105, while they lack expression of CD45 [6]. Moreover, ASCs have been shown to exert a wide range of immunomodulatory effects, such as inhibition of proinflammatory and induction of anti-inflammatory (IL-4, IL-13) cytokine secretion [7, 8]. The proregenerative properties of ASCs are mostly based on the secretion and intercellular transfer of extracellular membrane-derived vesicles (ExMVs), carrying growth factors including fibroblast growth factor (FGF), vascular endothelial growth factor (VEGF), and hepatocyte growth factor (HGF) [4, 9]. Moreover, ExMVs are able to transfer mRNA and miRNA, which after internalization into the host cell might affect its fate. Therefore, the unique properties of ASCs make them a great therapeutic tool not only in human medicine, but also veterinary medicine. However, the knowledge of the mechanisms responsible for survival and differentiation of ASCs is absolutely necessary to develop beneficial and innovative therapeutic strategies.

Recent alarming data suggest that age [4, 10–12], diseases [10, 11, 13–15], and lifestyle [12] seriously affect ASC regenerative and differentiation potential. Equine metabolic syndrome (EMS) is an increasingly frequently diagnosed endocrine disorder in the field of veterinary medicine [16]. The most characteristic features of EMS individuals include pathological obesity, abdominal and specific adiposity, fasting hyperinsulinemia, hyperglycemia, and insulin resistance (IR) [17, 18].

What is more, it was shown that the adipose tissue (AT) of EMS horses is characterized by hyperplasia and hypertrophy [19]. Increasing attention is paid to studying the impact of AT in the course of EMS, because AT is not only considered to be an energy storage tissue, but also a highly active endocrine organ. AT abundantly produces and releases adipokines, including leptin, resistin, retinol-binding protein 4, and visfatin. Moreover, excessive secretion of proinflammatory cytokines, such as interleukin 1 (IL-1), interleukin 6 (IL-6), and tumor necrosis factor alpha (TNF*α*) leads to local and systemic inflammation in EMS horses [20]. The proinflammatory microenvironment of adipose tissue negatively affects adipose-derived mesenchymal stromal stem cells (ASCs) that reside within it.

Our previous study showed that the potential for both chondrogenic differentiation and osteogenic differentiation of ASC isolated from EMS horses (ASC_EMS_) were seriously limited [10, 11]. We proposed that the rescue mechanism allowing those cells to maintain multipotency is autophagic flux. During autophagy, cytosolic components are first incorporated into autophagosomes and delivered to lysosomes in order to remove dysfunctional proteins or organelles. Autophagic removal of mitochondria has been termed mitophagy and is crucial for mitochondrial quality control. Selective removal of deteriorated mitochondria sustains cell survival and function. Autophagy is triggered during pathologic stresses, and it provides cells with building blocks for proteins and other biomolecules. For that reason, autophagy is a rescue mechanism, helping cells to survive in unfavorable conditions. It corresponds with our previous data as we observed increased autophagy in ASC_EMS_. Moreover, osteoblast and chondroblast precursors exhibited the impaired formation of extracellular matrix and the reduced expression of the following transcripts: bone morphogenetic protein 2 (BMP-2), vimentin, decorin, and Sox9. Obtained data suggested that under insulin resistance and EMS conditions, mitophagy is a predominant form of autophagy during chondrogenic differentiation of ASC_EMS_, and serves as a protective mechanism that allows maintaining multipotency. Increased autophagy and autophagic flux were also observed in the adipose tissue of obese humans and rodents [21, 22]. These exciting findings indicate the importance of autophagy in obesity development, however, the link between autophagy and/or selective mitophagy during adipogenic differentiation of ASC_EMS_ remains to be further defined. In the current study, we hypothesized that selective mitophagy may become a critical process in adipogenic differentiation.

The effectiveness of autologous ASC application in the course of EMS treatment strongly relies on their cytophysiological status, including viability, senescence, and accumulation of oxidative stress factors. EMS per se may be caused by profound changes in ASC physiology. Therefore, in this study, we investigated the impact of oxidative stress factors and mitochondrial deterioration in ASC_EMS_ on their adipogenic differentiation potential. We have found that mitochondrial and endoplasmic reticulum (ER) stress induces selective mitophagy and autophagy to protect ASC_EMS_ against cell death. Moreover, these factors directly contributed to the decreased differentiation potential of ASC_EMS._

## 2. Materials and Methods

All reagents used in this experiment were purchased from Sigma-Aldrich (Poland), unless indicated otherwise.

### 2.1. Experimental Animals

The study involved twelve mixed-sex, age-matched (9–14 years; mean ± SD, 11.2 ± 1.7 years) horses, divided into two groups: a group of individuals suffering from EMS (*n* = 6) and the control group, consisting of animals in good physical condition (*n* = 6). The division was done on the basis of detailed interviews with owners and clinical parameters such as body weight, body condition score, cresty neck score, combined glucose-insulin test, leptin concentration, and insulin levels. Comprehensive information about animals qualified to the experiments described in this paper are listed in Table 1.

### 2.2. Isolation and Culture of ASC

Adipose tissue samples were harvested from the tail base of experimental horses in accordance with ethical principles and surgical standards. For cell culture, the tissue fragments were washed with a sterile Hank's balanced salt solution (HBSS) supplemented with 1% of a penicillin, streptomycin, and amphotericin B (PSA) solution. Afterwards, the biological material was fragmented and digested with collagenase type I (1 mg/ml) for 40 minutes at 37°C. Cell suspension was centrifuged at 1200 ×g for 10 minutes at 23°C, then pellets were resuspended in the culture medium and transferred to the culture flasks.

Cultures were maintained under aseptic conditions at 37°C, 95% humidity, and 5% CO_2_. The cells were cultured in Dulbecco's modified Eagle's medium (DMEM) containing 4500 mg/l glucose supplemented with 10% of fetal bovine serum (FBS) and 1% of a penicillin, streptomycin, and amphotericin B (PSA) solution. Medium was changed every two days. After reaching 80–90% of confluence, the cells were passaged using a trypsin solution (TrypLE Express, Life Technologies).

### 2.3. Immunophenotyping

To determine the phenotype of the cells, the expression of the following surface markers was investigated: CD44, CD45, and CD90. The cells were prepared for flow cytometry as follows: ASCs were treated with TrypLE Express solution, then extensively washed with HBSS, and resuspended at a total of 5 × 105 cells/ml. Afterwards, cell suspension was incubated with antibodies preconjugated with peridinin chlorophyll protein (PerCP) and fluorescein isothiocyanate (FITC) (anti-CD45, Novus Biologicals, NB1006590APC; anti-CD44, R&D Systems, MAB5449; and anti-CD90, Abcam, ab225) or appropriate isotope control antibodies at 4°C for 20 minutes. The analysis was performed using Becton Dickinson FACS Calibur Flow Cytometer. The results were analyzed by CellQuest Pro software (Becton Dickinson, Franklin Lanes, New Jersey, USA).

Additionally, cell cycle analysis was performed using Muse Cell Cycle Assay Kit with Muse Cell Analyzer (Merck) according to the manufacturers' instruction.

### 2.4. Assessment of ASC Proliferation Rate

The viability of cultured cells was evaluated using the resazurin-based assay kit (TOX8). To perform the experiment, cells were seeded on a 24-well plate at a concentration of 1 × 10^4^ cells per cm^2^. The proliferation rate was determined at the 1st, 2nd, and 5th day of the experiment. Absorbance levels were measured spectrophotometrically (SPECTROstar Nano, BMG LABTECH, Germany) at a wavelength of 690 nm as a reference wavelength and 600 nm for resazurin. Additionally, the population-doubling time (PDT) was estimated using the formula presented below:

(1) $$\begin{array}{c} PDT=\frac{\left(duration\times \log\left(2\right)\right)}{\left(\log\left(final\;concentration\right)-\log\left(initial\;concentration\right)\right)}. \end{array}$$

The synthesis of DNA in ASC cultures was evaluated by the detection of 5-bromo-2-deoxyuridine incorporation into cellular deoxyribonucleic acid. Cell proliferation rates were measured twice—after 24 and 120 hours of culture. The experiment was conducted using bromodeoxyuridine (BrdU) assay (Abcam). The absorbance was measured with a spectrophotometer microplate reader at a wavelength of 450/550 nm.

For the clonogenic assay, cells were seeded on 6-well culture plates at a density of 110 cells per cm^2^. After 5 days, biological material was fixed with 4% ice-cold paraformaldehyde and stained with pararosaniline. Colonies consisting of 50 or more cells were considered as colony forming units (CFU). The experimental procedure was performed in triplicate. The efficiency of CFU was calculated according to the following formula:

(2) $$\begin{array}{c} \frac{Number\text{ of }colonies>50\;cells}{Number\text{ of }seeded\;cells}\times 100\%. \end{array}$$

All experimental procedures included in the proliferation assay were performed in accordance with the manufacturer's instructions.

### 2.5. Adipogenic Differentiation of ASC

Cell differentiation was induced using an adipogenic differentiation medium containing 0.5 mM of 1-methyl-3-isobutylxantine (IMBX), 0.5 mM of dexamethasone, 6.25 *u*g/ml of insulin, 60 *u*M of indomethacin, 50 *u*g/ml of gentamicin, 10% of FBS, and Dulbecco's modified Eagle's medium (DMEM) with Nutrient F-12 Ham. The cells were seeded at a density of 15 × 10^3^ cells per cm^2^ on 24-well plates, and the culture media were changed every second day. Cells cultured in standard growth medium were used as a control for the experiment and allowed to establish differentiation effectiveness. Stimulation toward adipogenic lineage lasted 10 days. To evaluate the effects of the adipogenesis stimulation, the cells were fixed with 4% PFA. Then, Oil Red O staining was used for the detection of intracellular lipid droplets. The results obtained during the staining procedure were analyzed using an Axio Observer A1 inverted microscope (Zeiss, Germany), while the photographic documentation was made using a Canon PowerShot digital camera. The percentage of the Oil Red O stained area was calculated using Image J.

### 2.6. Examination of ASC Morphology and Ultrastructure

Cell morphology was evaluated using an inverted epifluorescent microscope (Zeiss, Axio Observer A.1) as described elsewhere [20]. In order to perform the observations, cells were fixed with 4% PFA overnight at 4 °C. Biological material was rinsed with HBSS, then the cell membranes were permeabilized with 0.1% Triton X-100 solution for 15 minutes at room temperature. To visualize actin filaments, the cells were stained with atto-594-labelled phalloidin diluted 1 : 800 in HBSS for 40 minutes under protection from light. Nuclei were imaged using diamidino-2-phenylindole (DAPI) diluted 1 : 1000 in HBSS, and the cells were incubated with DAPI for 5 minutes at room temperature. Mitochondria were stained with Mito Red fluorescence dye, while endoplasmic reticulum was visualized using a commercially available kit—CellLight ER-RFP, BacMam 2.0 (Life Technologies). All images were captured with a Canon PowerShot camera. Photographs obtained as a result of DAPI/phalloidin staining were merged using AxioVision 4.8 software (Carl Zeiss, Jena, Germany). Excluding Mito Red staining, all describing analyses were performed after the 5th (noninduced cells) and 10th (cells after induction of adipogenesis) day of culture.

Detailed morphological analyses were conducted using a scanning electron microscope (SEM, Zeiss EVO LS15) and focused ion beam (FIB, Zeiss, Cobra, AURIGA 60). For scanning electron microscopy analysis, the cell cultures were fixed with 2.5% glutaraldehyde for 1 h at room temperature, then dehydrated in graded ethanol-water mixtures, air-dried for 30 minutes at room temperature, and coated with gold (ScanCoat 6, Oxford). Prepared samples were captured using a SE1 detector at 10 kV filament tension. For transmission electron microscopy analysis, the samples were fixed overnight at 4°C in 2.5% glutaraldehyde. Afterwards, the cells were rinsed several times with PBS and incubated for 2 h with 1% osmium tetroxide. The biological material was then contrasted with lead citrate and uranyl acetate, dehydrated in a graded series of acetone, and embedded using an Agar Low Viscosity Resin Kit (Agar Scientific Ltd., Essex, UK). Ultrathin sections were obtained using an ultramicrotome (Leica EM UC7) and collected on copper grids. The cells were observed with a TEM detector at 20 kV filament tension. The cells for FIB imaging were centrifuged and fixed with 2.5% glutaraldehyde. The procedure of preparing the biological material for FIB was conducted as previously described by Marycz et al. [10]. The imaging was conducted using an SE2 detector at 2 kV of electron beam voltage.

### 2.7. Immunofluorescence Stainings

In order to perform the assay, cultures were fixed with 4% PFA for 40 minutes. After fixation, cells were extensively washed with HBSS. Washing step was repeated three times. Then, the cell membranes were permeabilized for 20 minutes at room temperature using 0.5% Triton X-100 and rinsed with HBSS once again. After this procedure, unspecific binding sites were blocked with blocking buffer composed of HBSS supplemented with 10% goat serum and 0.2% Tween-20 for 35 minutes. Then, the cells were incubated overnight with specific primary antibodies against Ki67 (Abcam) diluted 1 : 200 in HBSS with 1% goat serum and 0.2% Tween-20. Following procedure, the biological material was washed with HBSS and incubated with mouse anti-rabbit secondary antibodies conjugated with atto-594 and atto-488 (dilution 1 : 200) for 1 hour in the dark. Nuclei were counterstained by incubation with DAPI for 5 min.

To image 5-methylocytosine (5-MC) and 5-hMC, cells were fixed with 4% PFA for 40 minutes and permeabilized with 0.5% Triton X-100 in HBSS. Afterwards, the cells were incubated with 4 N HCl for 15 min, then with blocking buffer containing HBSS with 10% goat serum and 0.2% Tween-20 for 45 minutes. Incubation with primary antibodies (anti-5-methylocytosine, ab73938, Abcam; anti-5-hydroxymethylocytosine, ab106918, Abcam) diluted 1 : 200 in HBSS supplemented with 1% of goat serum and 0.2% Tween-20 was conducted overnight. After three washes with HBSS, the cells were treated with specific secondary antibodies (goat anti-rat, ab150157, Abcam; goat anti-mouse, ab150113, Abcam) conjugated with atto-488 diluted 1 : 200 for 1 hour.

Immunofluorescent staining of investigated cells was also performed using anti-LAMP2 and anti-PARKIN antibodies (ab25631, ab77924; Abcam). The cells were observed and imaged using confocal microscopy (Observer Z1 Confocal Spinning Disc V.2, Zeiss).

### 2.8. Oxidative Stress and Senescence Assay

The procedure of oxidative stress level quantification was performed after 10 days of adipogenesis stimulation comparing with control, noninduced cell cultures. Superoxide dismutase (SOD) activity analysis was conducted using a SOD Assay Kit, and the level of nitric oxide (NO) was assessed with a Griess reagent kit (Life Technologies, USA), whereas reactive oxygen species (ROS) concentration was evaluated in two ways: with a 5-(and-6)-chloromethyl-2′,7′-dichlorodihydrofluorescein diacetate (H2DCF-DA, Life Technologies) solution and using a Muse® Cell Cycle Assay Kit with a Muse Cell Analyzer (Merck). Detection of senescence-associated *β*-galactosidase activity was performed using the Senescence Cells Histochemical Staining Kit. The number of viable and dead cells were estimated with a Cellstain Double Staining Kit. The dead cells' nuclei were stained with propidium iodide, while the nuclei of viable cells were dyed with Calcein-AM. Images of colored cell organs were captured using a Canon PowerShot camera. All procedures were carried out based on the protocols provided by the manufacturers.

### 2.9. Analysis of Gene Expression: Real-Time Reverse Transcription Polymerase Chain Reaction (qRT-PCR)

After 120 hours of culture, cells were rinsed gently using HBSS and homogenized with 1 ml of TRI Reagent. Total RNA was isolated from cells after culture, and tissue samples were collected directly from experimental animals according to a single-step method described previously by Chomczynski and Sacchi [23] and RT-PCR was performed as described elsewhere [24]. The received RNA diluted in DEPC-treated water was analyzed for quantity and quality by means of a nanospectrophotometer (WPA Biowave II). Enzymatic digestion of genomic DNA (gDNA) and complementary DNA (cDNA) synthesis was conducted using a Takara PrimeScript™ RT Reagent Kit with gDNA Eraser (Perfect Real Time) following the manufacturer's protocol. For each reaction, 150 ng of total RNA was used. Both genomic DNA digestion and cDNA synthesis were performed using a T100 Thermal Cycler (Bio-Rad). The qRT-PCR reactions were carried out using a SensiFAST SYBR Green Kit (Bioline). Each reaction mixture contained 2 *μ*l of accurate matrix in a total volume of 20 *μ*l. Primer concentration was 0.5 *μ*M. Sequences of the primers used in the quantitative real-time PCR are listed in Table 2. All qRT-PCR reactions were conducted with CFX Connect™ Real-Time PCR Detection System (Bio-Rad). The value of the threshold cycle (Ct) was used to calculate the fold change in relation to the expression of the GAPDH housekeeping gene.

### 2.10. Statistical Analysis

The results were evaluated based on measurements obtained in subsequent repetitions. All experiments were performed at least in triplicate. Differences between groups were determined using the nonparametric *t*-test. Statistical analysis was performed with GraphPad Prism 5 software (La Jolla, USA). Differences were considered statistically significant for *p* < 0.05.

## 3. Results

### 3.1. Immunophenotyping and Multipotency Assay

**Figure 1:**
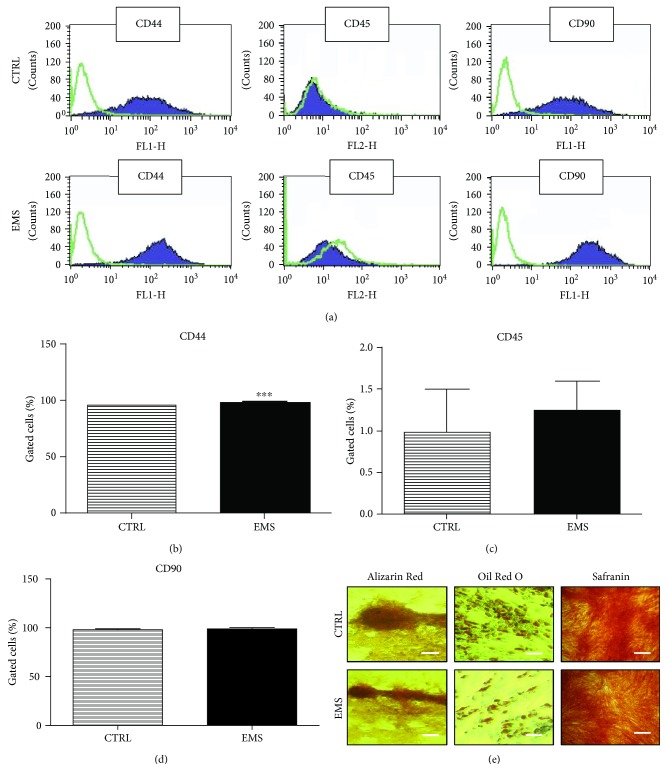
Immunophenotyping and multipotency assay. Representative histograms from flow cytometry analysis, green lines—isotope controls and purple lines—specific for surface antigen antibodies (a). Cells were analyzed for the expression of CD44 (b), CD45 (c), and CD90 (d). Moreover, to confirm the multipotency of isolated cells, they were induced into osteogenic, adipogenic, and chondrogenic lineages. Effectiveness of differentiation was confirmed by specific staining (e). Results were expressed as mean ± SD. Scale bar 100 *μ*m. ^∗∗∗^*p* < 0.001.

Isolated cells presented typical for the ASC profile of surface antigens (Figure 1(a)). They expressed CD44 (Figure 1(b)) and CD90 (Figure 1(c)) while lacking the expression of the CD45 hematopoietic marker (Figure 1(d)). Moreover, to confirm the multipotent properties of isolated cells, they were cultivated into osteogenic, chondrogenic, and adipogenic medium. The effectiveness of differentiation was confirmed by specific stainings (Figure 1(e)). The accumulation of mineralized matrix was proved by Alizarin Red, while the formation of proteoglycans was proved by Safranin O dye. Intracellular lipid droplets were stained with Oil Red O.

### 3.2. Growth Kinetics and Morphology of ASC Cultured in Control Conditions

**Figure 2:**
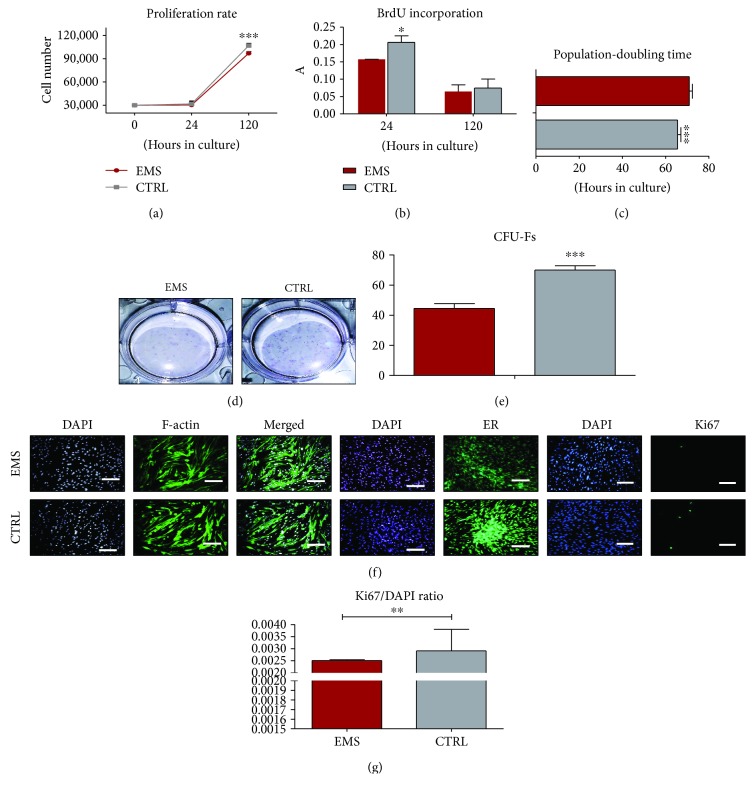
Growth kinetics of ASCs cultured in control conditions. Cell number estimated using a resazurin-based assay (a) and incorporation of BrdU (b). Population-doubling time was calculated using the number of cells in each of the experimental time points (c). The ability of cells to form colonies originating from one cell was evaluated by CFU-F assay. Representative photographs showing colonies stained with pararosaniline (d) and quantitative data obtained by the application of a CFU-F algorithm (e). The morphology of cells was investigated using fluorescence staining for f-actin and endoplasmic reticulum (ER) (f). Moreover, proliferation of cells was estimated by immunofluorescence staining for the Ki67 antigen as presented on representative images. Data was quantified and the Ki67/DAPI ratio was calculated (g). Results are expressed as mean ± SD. Scale bar 100 *μ*m. ^∗^*p* < 0.05, ^∗∗^*p* < 0.01, and ^∗∗∗^*p* < 0.001.

The viability and proliferation rate of investigated cells were established during a five-day lasting culture period. The number of metabolic-active cells was established with a resazurin-based assay (TOX8) in accordance with the manufacturer's protocol. Cells from both investigated groups proliferated at a similar rate during the experiment. Only on the last day did the ASC_EMS_ proliferate at a significantly lower rate in comparison to control cells (Figure 2(a), *p* < 0.001). Furthermore, we evaluated the rate of proliferating cells in culture by the analysis of BrdU incorporation after 24 and 120 hours of culture (Figure 2(b)). ASC_CTRL_ was characterized by a significantly increased proliferation rate only after 24 hours of cultivation (*p* < 0.05). PDT was estimated based on the number of cells in each day of experiment (Figure 2(c)). The results indicated that ASC_EMS_ required more time to double their number in comparison to control cells (*p* < 0.001). The ability of cells to form colonies originating from single cells was performed using the CFU-F assay. For that reason, colonies were stained with pararosaniline (Figure 2(d)) and counted under a light microscope. The greatest number of colonies is clearly visible in ASC_CTRL_. Furthermore, data was quantified using a CFU-F algorithm (Figure 2(e)), revealing the reduction of CFU-F in the ASC_EMS_ group (*p* < 0.001). Cell morphology was evaluated on the last (5th) day of the experiment using staining for f-actin and ER (Figure 2(f)). An enlarged cell body and the occurrence of stress fibers were noted in ASC_EMS_ simultaneously with decreased fluorescence intensity coming from ER which indicates its decreased activity and/or impairment. Moreover, by the application of immunofluorescence staining, the accumulation of Ki67 in cell nuclei was evaluated (Figure 2(f)). Data from representative photographs was quantified by calculating the ratio of Ki67-positive cells to the total number of nuclei (Figure 2(g)). Obtained results confirmed a decreased proliferation of ASC_EMS_ in comparison to the control group (*p* < 0.01).

### 3.3. Growth Kinetics and Morphology of ASC_CTRL_ and ASC_EMS_ Cultured under Adipogenic Conditions

**Figure 3:**
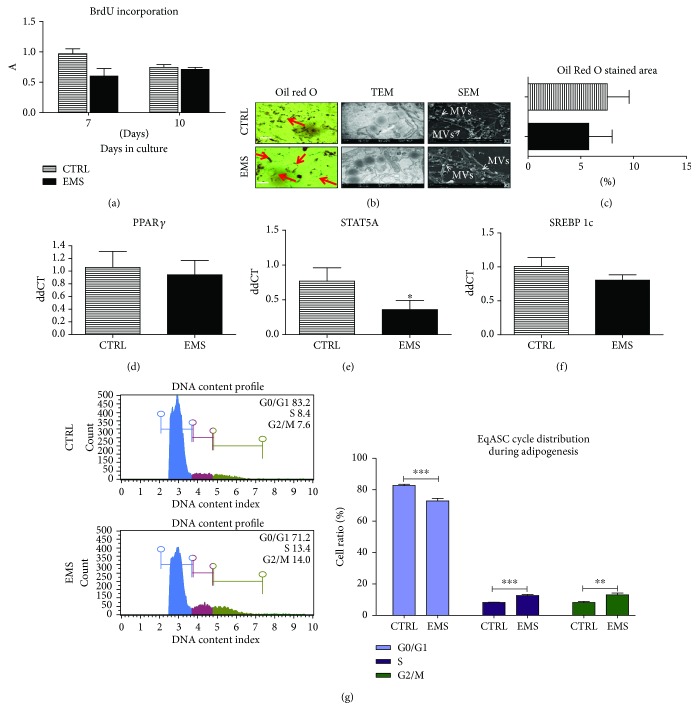
Growth kinetics and morphology of ASC_CTRL_ and ASC_EMS_ cultured under adipogenic conditions. To evaluate the proliferation rate under adipogenic differentiation, incorporation of BrdU was established (a) in both of the investigated groups. To confirm adipogenesis and establish its effectiveness, cells were stained with Oil Red O. Moreover, the secretion of MVs was evaluated using SEM imaging while the ultrastructure of cells was visualized using a TEM microscope (b). The Oil Red O-stained area was quantified using Image J (c). Using RT-PCR, expression of PPAR*γ* (d), STAT5A (e), and SREBP 1c (f) was established. Cell cycle (DNA content) was analyzed in both of the investigated cultures using a cell analyzer (g). Obtained results indicate a more effective differentiation in control cells as they stop to proliferate in order to differentiate into adipocytes. ASC isolated from EMS individuals seems to be more resistant to adipogenic stimuli. Results are expressed as mean ± SD. Scale bar 200 *μ*m. ^∗^*p* < 0.05, ^∗∗^*p* < 0.01, and ^∗∗∗^*p* < 0.001.

**Figure 4:**
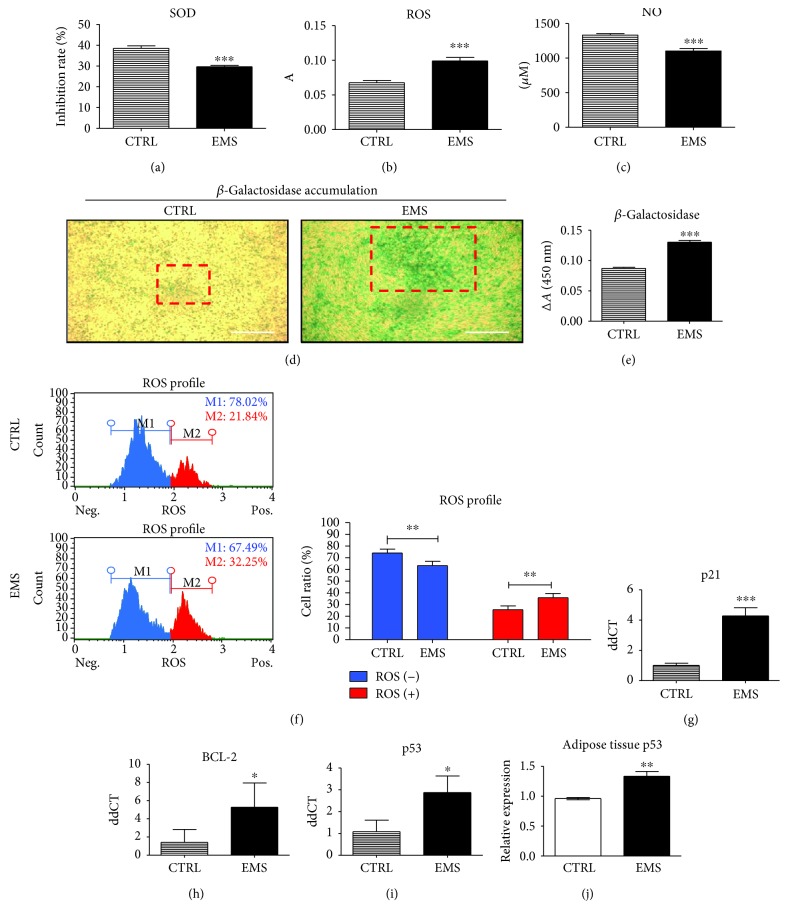
Assessment of oxidative stress factors and apoptosis under adipogenic conditions. To evaluate the levels of oxidative stress, the amount of SOD (a), ROS (b), and NO (c) in culture supernatants were assessed. Representative photographs showing the results of senescence-associated *β*-galactosidase staining. Boxed regions with red edges indicate the regions with excessive *β*-galactosidase accumulation. Accumulation of *β*-galactosidase was also quantified by spectrophotometric measurement of dye reduction (e). Intracellular accumulation of ROS was assessed with a cell analyzer (f). Moreover, the expression of the following transcripts was investigated using the RT-PCR method: p21 (g), BCL-2 (h), and p53 (i). mRNA levels of p53 was also analyzed in adipose tissue homogenates of healthy and EMS diagnosed horses (j). Results are expressed as mean ± SD. Scale bar 250 *μ*m. ^∗^*p* < 0.05, ^∗∗^*p* < 0.01, and ^∗∗∗^*p* < 0.001.

Proliferation of cells was established by the BrdU incorporation assay (Figure 3(a)). However, no statistical differences were observed among the investigated cells neither on the 7th day nor on the 10th. Effectiveness of differentiation into adipocytes was visualized by applying Oil Red O staining (Figure 4(b)). Representative photographs showed an increased number of lipid droplets in ASC_CTRL_, although these were smaller in size. Lipid droplets were also visualized by a TEM microscope. Ultrathin sections by TEM showed large, electron-dense lipid droplets in ASC_EMS_, which sometimes were associated with mitochondria. ASC_CTRL_ had lipid droplets that resembled those in ASC_EMS_, however, these were much less electron dense. Those differences in lipid droplet features may indicate a distinct lipid composition and thus, the energy status of the cells. Using SEM, secretion of MVs by cells was analyzed but no visible differences were noted. Oil Red O staining was quantified using Image J (Figure 4(c)), however no differences were observed. The expression of PPAR*γ* (Figure 4(d)), STAT5A (Figure 4(e)), and SREBP 1c (Figure 4(f)) was established. Only STAT5A expression showed a statistically significant difference as its mRNA was downregulated in ASC_EMS_ (*p* < 0.05). Analysis of DNA content revealed an increased number of cells in the G0/G1 phase in the control group (*p* < 0.001) (Figure 4(g)). However, an increased number of cells in the S and G2/M phases were noted in ASC_EMS_ (*p* < 0.001 and *p* < 0.01, resp.).

### 3.4. Assessment of Oxidative Stress Factors and Apoptosis under Adipogenic Conditions

After the last (10th) day of adipogenesis, the SOD (Figure 4(a)), ROS (Figure 4(b)), and NO (Figure 4(c)) levels were evaluated. The activity of SOD was significantly downregulated in ASC_EMS_ (*p* < 0.001), while the amount of ROS (*p* < 0.001) was upregulated. Interestingly, the amount of NO was decreased in ASC_EMS_ (*p* < 0.001). Representative photographs of *β*-galactosidase staining are shown on Figure 4(d). Data was also quantified by the measurement of the dye reduction indicating increased ASC_EMS_ senescence. Moreover, the accumulation of intracellular ROS was analyzed using a Muse Cell Analyzer (Figure 4(f)) indicating increased ROS levels in ASC_EMS_ (*p* < 0.01). Expression of apoptotic-related genes was established using RT-PCR. The mRNA levels of p21 (Figure 4(g)) were upregulated in ASC_EMS_ (*p* < 0.001) while BCL-2 was upregulated (Figure 4(h)). Expression of p53 was increased in ASC_EMS_ (*p* < 0.05) (Figure 4(i)). Expression of p53 was also evaluated in adipose tissue of horses as it stands as a stem cell niche (Figure 4(j)). Similarly, p53 expression was upregulated in adipocytes from EMS-suffering individuals (*p* < 0.01).

### 3.5. Epigenetic State of ASC_CTRL_ and ASC_EMS_ Cultured under Adipogenic Condition

**Figure 5:**
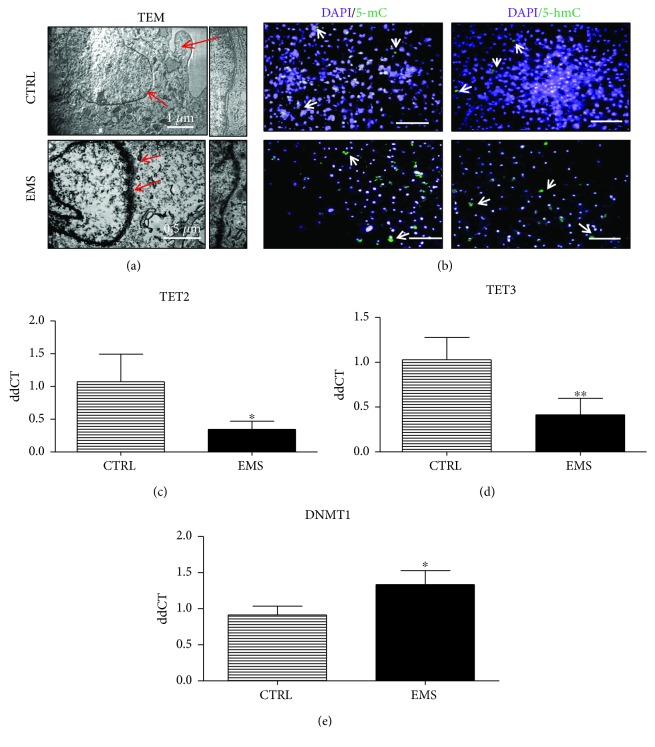
Epigenetic state of ASC_CTRL_ and ASC_EMS_ cultured under adipogenic conditions. TEM images of nuclei (a) and their higher magnification showing heterochromatin underneath the nuclear envelope. ASC_EMS_ was characterized by a breakdown of heterochromatin associated with the inner nuclear membrane. Immunofluorescence staining for genomic distribution of 5-mC and 5-hmC (b) was performed in order to evaluate DNA methylation status. Using RT-PCR, expression of TET2 (c), TET3 (d), and DNMT1 (e) was evaluated. Obtained results indicate increased DNA methylation in ASC_EMS_. Results are expressed as mean ± SD. Scale bar 250 *μ*m. ^∗^*p* < 0.05 and ^∗∗^*p* < 0.01.

Nuclei of cells were visualized using a TEM microscope (Figure 5(a)). ASC_EMS_ were characterized by the loss of heterochromatin amount at the nuclear periphery. Furthermore, DNA methylation status was analyzed by immunofluorescence staining for 5-mC and 5-hmC (Figure 5(b)). Obtained results indicate an increased methylation and decreased hydroxymethylation in ASC_EMS_. Moreover, expression of TET2 (Figure 5(c)) and TET3 (Figure 5(d)) genes was investigated using RT-PCR. The amount of both genes' mRNA was downregulated in ASC_EMS_ (*p* < 0.05 and *p* < 0.01, resp.) confirming staining results. Expression of DNMT-1 was upregulated (*p* < 0.05) in ASC_EMS_.

### 3.6. Assessment of Autophagy in ASC_CTRL_ and ASC_EMS_ Cultured under Adipogenic Condition

**Figure 6:**
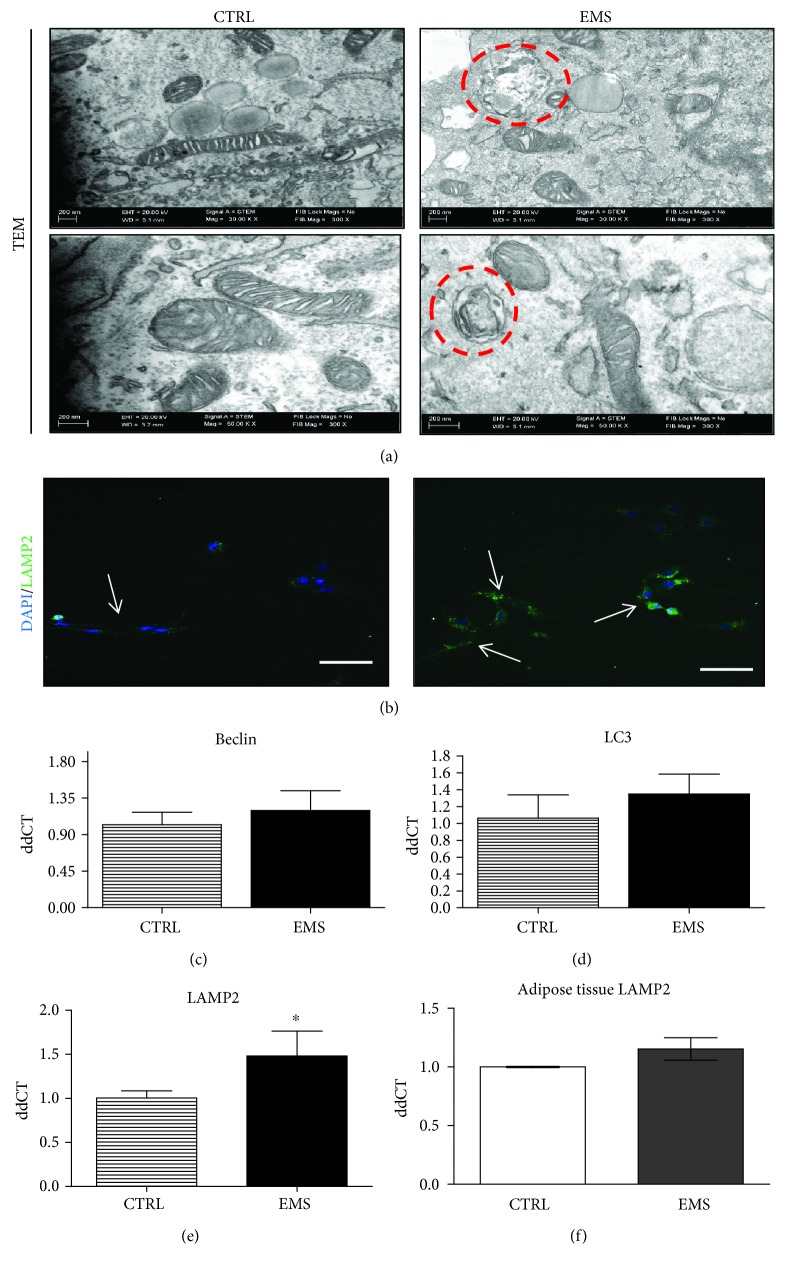
Assessment of autophagy in ASC_CTRL_ and ASC_EMS_ cultured under adipogenic conditions after the 10th day. Formation of autophagosomes and autolysosomes was visualized using TEM imaging. Circles with red edges indicate autophagosomes within the cell (a). Moreover, using immunofluorescence staining, LAMP2 localization was investigated under a confocal microscope (b). Using RT-PCR, the expression of autophagy-related genes including Beclin (c), LC3 (d), and LAMP 2 (e) was evaluated. Upregulation of LAMP2 indicates autolysosome formation which is required to complete the autophagic removal of damaged organelles. Interestingly, no differences in LAMP2 expression in adipose tissue (f) was observed. Results are expressed as mean ± SD. Scale bar 250 *μ*m. ^∗^*p* < 0.05.

Formation of autophagosomes in cells was visualized using a TEM microscope. Representative photographs (Figure 6(a)) indicated an increased autophagosome formation in ASC_EMS_. Localization of LAMP2 in cells was established using immunofluorescence staining and confocal microscope (Figure 6(b)). Obtained pictures indicated an increased LAMP2 accumulation in ASC_EMS_. Expression of autophagy-related genes was investigated using RT-PCR: Beclin (Figure 6(c)), LC3 (Figure 6(d)), and LAMP2 (Figure 6(e)). However, only the expression of LAMP2 showed statistically significant differences between groups as it was upregulated in ASC_EMS_ (*p* < 0.05), confirming confocal microscope data. Interestingly, no differences in LAMP2 expression in adipose tissue of horses were noted (Figure 6(f)).

### 3.7. Mitochondrial Dynamics and Clearance in ASC_CTRL_ and ASC_EMS_ Cultured under Adipogenic Conditions

**Figure 7:**
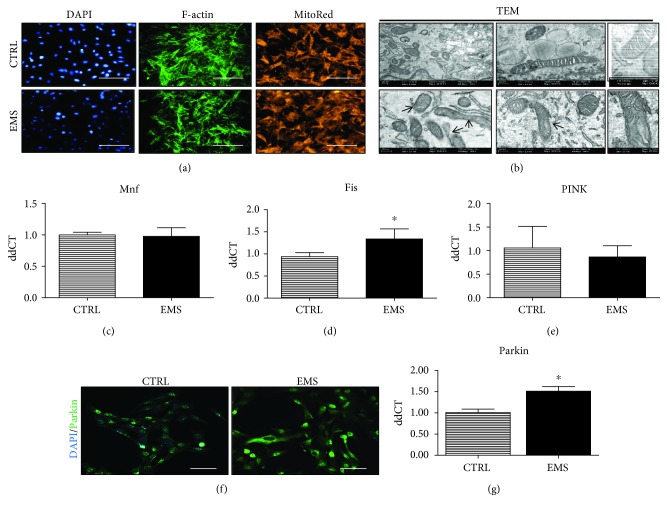
Mitochondrial dynamics and clearance in ASC_CTRL_ and ASC_EMS_ cultured under adipogenic conditions. Using fluorescence staining, cells' cytoskeleton (f-actin) and mitochondrial net were visualized (a). Moreover, a TEM microscope allowed for a deeper assessment of mitochondrial morphology (b). Mitochondria from ASC_CTRL_ presented typical, elongated morphology, while ASC_EMS_ cells were characterized by mitochondrial aberrations including membrane and cristae raptures. Moreover, mitochondrial dynamics was assessed by RT-PCR as the expression of Mnf (c) and Fis (d) genes was investigated. However, no differences were observed between groups. Similarly, no differences in PINK expression were noted (e). To evaluate the amount of Parkin protein, immunofluorescence staining and RT-PCR were performed. Representative photographs obtained from a confocal microscope indicated increased Parkin accumulation in ASC_EMS_ (f). Those data were also confirmed by the analysis of Parkin mRNA levels, as it was significantly upregulated in ASC_EMS_ (g). Results are expressed as mean ± SD. Scale bar 250 *μ*m. ^∗^*p* < 0.05.

Actin cytoskeleton and mitochondrial distribution within it was investigated using fluorescent stainings (Figure 7(a)). Fluorescence intensity of phalloidin and Mito Red was decreased in ASC_EMS_ as showed on representative photographs. Moreover, morphology of mitochondria was visualized using a TEM microscope (Figure 7(b)). Mitochondria in ASC_CTRL_ were characterized by a typical elongated morphology, while mitochondria in ASC_EMS_ suffered from morphological impairment including membrane raptures and disarrayed cristae. Furthermore, mitochondrial dynamics was assessed by investigation of Mnf (Figure 7(c)), Fis (Figure 7(d)), and PINK (Figure 7(e)) expression. However, no significant changes in their mRNA levels were noted. Accumulation of Parkin in cells was analyzed by immunofluorescent staining and confocal microscope imaging (Figure 7(f)). The fluorescence intensity of Parkin was increased in ASC_EMS_ which stands in good agreement with RT-PCR results confirming upregulation of Parkin in ASC_EMS_ (*p* < 0.05) (Figure 7(g)).

### 3.8. Endoplasmic Reticulum Stress in ASC_CTRL_ and ASC_EMS_ Cultured under Adipogenic Condition after Day 10

**Figure 8:**
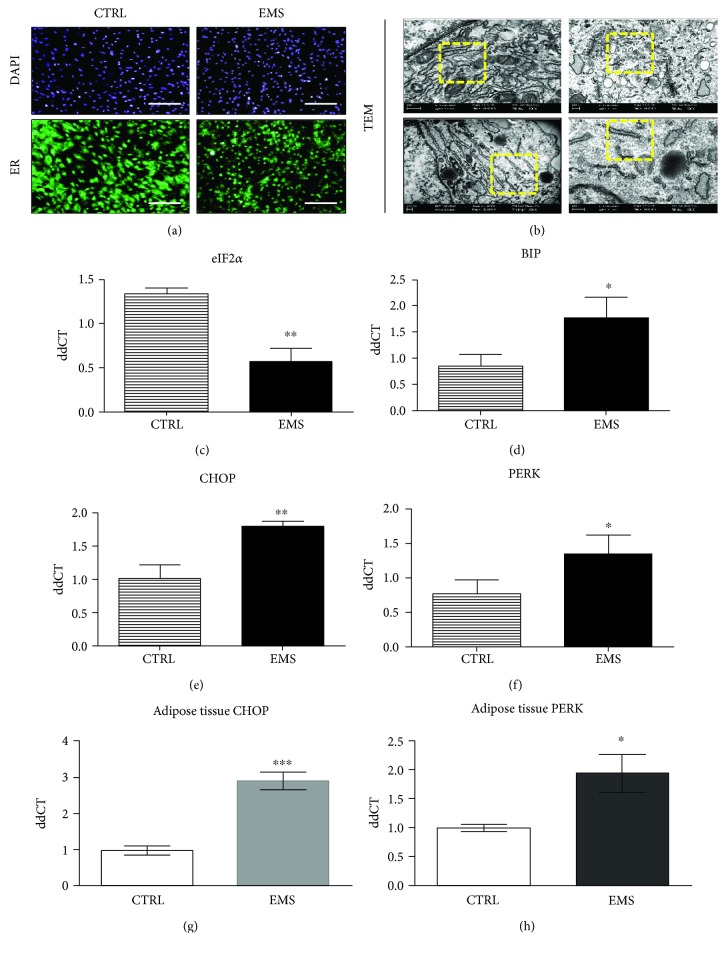
Endoplasmic reticulum stress in ASC_CTRL_ and ASC_EMS_ cultured under adipogenic conditions after the 10th day. ER condition and distribution were visualized under fluorescent and TEM microscopes. The more intense fluorescence ER signal was noted in ASC_CTRL_, where ER was robustly expanded throughout the cells (a). In the case of ASC_EMS_, ER was fragmented and disintegrated as observed under TEM (b). To further investigate ER stress in investigated cells, we establish the expression of eIF2*α* (c), BIP (d), CHOP (e), and PERK (f). Obtained results indicated an induction of unfolded protein response and ER stress in ASC_EMS_. Furthermore, we decided to evaluate ER stress in ASC niche-adipose tissue. Interestingly ER stress was also observed within adipocytes of EMS horses as increased expression of both CHOP (g) and PERK (h) transcripts was noted. Results are expressed as mean ± SD. Scale bar 250 *μ*m. ^∗^*p* < 0.05, ^∗∗^*p* < 0.01, and ^∗∗∗^*p* < 0.001.

Using fluorescence staining we evaluated the ER distribution in cells (Figure 8(a)). Decreased fluorescent ER signal was observed in ASC_EMS_ which indicates its decreased activity and/or impairment. TEM photographs confirmed ER deterioration in ASC_EMS_ as it was fragmented and swollen (Figure 8(b)). On the contrary, ASC_CTRL_ were characterized by well-developed ER with numerous ribosomes. To further analyze ER stress, expression of eIF2*α* (Figure 8(c)), BIP (Figure 8(d)), CHOP (Figure 8(e)), and PERK (Figure 8(f)) was investigated by RT-PCR. eIF2*α* was downregulated (*p* < 0.01) in ASC_EMS_ while the remaining transcripts were upregulated. Furthermore, expression of both CHOP (Figure 8(g)) and PERK (Figure 8(h)) was evaluated in adipose tissue of qualified horses. Both transcripts were upregulated in EMS horses' adipocytes (*p* < 0.001 and *p* < 0.05, resp.).

### 3.9. Inflammation and Insulin Sensitivity

**Figure 9:**
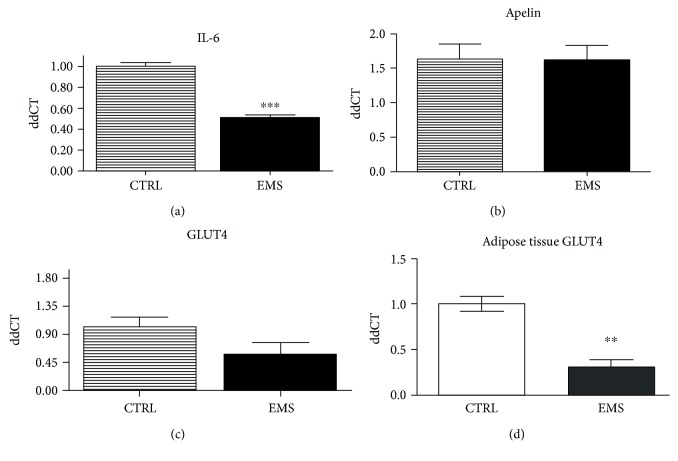
Inflammation and insulin sensitivity. Using RT-PCR, the expression of IL-6 (a), apelin (b), and GLUT4 (c) was assessed in ASCs. Moreover, GLUT-4 levels were also established in adipose tissue of horses (d). Interestingly, no differences in GLUT4 expression were observed in ASC; however, it was significantly downregulated in adipose tissue of EMS horses. Results are expressed as mean ± SD. Scale bar 250 μm. ^∗∗^*p* < 0.01 and ^∗∗∗^*p* < 0.001.

Expression of IL-6 was downregulated in ASC_EMS_ as indicated by RT-CPR results (Figure 9(a)). Expression of both apelin (Figure 9(b)) and GLUT4 (Figure 9(c)) did not show significant differences between groups. However, GLUT4 expression in adipose tissue was downregulated in EMS horses' adipocytes indicating decreased insulin sensitivity (Figure 9(d)).

## 4. Discussion

Until recently, adipose tissue (AT) has been considered only as an energy storage, but in fact it is an active endocrine organ, playing a crucial role in EMS development [19]. At the same time, it is a source of ASCs, an extensively applied cell population in the field of equine regenerative medicine. However, the unfavorable microenvironment of AT (chronic inflammation) in EMS horses strongly affects cytophysiological properties of ASCs residing within it. In this study, we showed that ASC_EMS_ cultured in the control medium suffered from impaired proliferative activity, viability, reduced number of Ki67-positive cells, prolonged population-doubling time, and reduced clonogenic potential. Interestingly, we have observed an increased expression of the CD44 surface marker, which is strongly correlated with inflammation, insulin resistance, and type 2 diabetes. It was found that CD44 knockout mice were protected against obesity and insulin resistance development [25]. Moreover, it was demonstrated that the application of the CD44 monoclonal antibody protected mice fed with a high-fat diet against insulin resistance, liver steatosis, and hyperglicemia [26]. Thus, the upregulation of CD44 surface marker expression in ASC_EMS_ can become a diagnostic marker in EMS diagnosis and treatment.

Furthermore, we investigated growth kinetics, mitochondrial dynamics, autophagy, and apoptosis during adipogenic differentiation of ASCs in order to assess metabolic changes that occur during this process. Impaired proliferative activity along with decreased viability and enhanced apoptosis were also observed in ASC_EMS_ under adipogenic differentiation conditions. We found that ASC_EMS_ accumulated smaller lipid droplets, and the expression of adipogenesis-related genes was decreased in those cells in comparison to the control group. Moreover, ASC_EMS_ showed the following characteristics during adipogenic differentiation: (i) senescent phenotype, (ii) increased aging, (iii) excessive accumulation of oxidative stress factors, and ultimately, (iv) apoptosis.

Adipogenesis is a well-orchestrated process, in which preadipocyte proliferation, differentiation, and lipid biosynthesis are regulated by a set of factors that include signal transducer and activator of transcription 5A (STAT5A), sterol-regulatory-element-binding protein 1c (SREBP 1c), and peroxisome proliferator-activated receptor gamma (PPAR*γ*). Here, we observed that ASC_EMS_ under adipogenic conditions exhibited lower proliferative and metabolic activity when compared to healthy ASCs. This can be explained by the observed downregulation of genes involved in early events of adipocyte differentiation—STAT5A and SREBP1c. It was found that the expression of STAT5A and SREBP1c in subcutaneous adipose tissue was significantly reduced in type 2 diabetic patients [27]. A comprehensive study using a mice model showed decreased expression of PPAR*γ* in adipose tissue of diabetic mice versus healthy wild type donors [28]. In turn, STAT5A was shown to be involved in adipocyte differentiation probably by controlling the expression of PPAR*γ*, and type 2 diabetic patients had decreased expression of both PPAR*γ* and STAT5A genes [27]. Interestingly, only decreased expression of STST5A was observed in ASC_EMS_, which indicated a different mechanism underlying the deterioration of stem cells caused by metabolic syndrome from those observed in adipose tissue. The obtained data are consistent with the findings of other groups [29–31] and clearly confirm that the impairment of adipogenesis is linked to metabolic syndrome and the development of insulin resistance. Moreover, Oil Red O staining and focused ion beam microscopy (FIB) revealed different sizes of lipid droplets between ASC_CTRL_ and ASC_EMS_. We have observed that ASC_EMS_ accumulates lipid droplets (LD) of various sizes under adipogenic conditions in contrast to ASC_CTRL_ where lipid droplet sizes were similar. Interestingly, Yang et al. have recently shown that adipocyte size variation along with the presence of large adipocytes are predictors of insulin resistance and type 2 diabetes [32].

Our results confirm the thesis of a niche effect on the metabolic status of stem cells residing within it. The adverse microenvironment of EMS horses' adipose tissue negatively affects ASC multipotency and metabolism. Here, we observed an excessive accumulation of ROS and reduced level of SOD in ASC_EMS_ during adipogenic differentiation. The extent of adipogenesis promoting nitric oxide was reduced in ASC_EMS_, which may be partially responsible for the impaired differentiation in these cells. Moreover, Minamino et al. showed, that calorie overload in mice with type 2 diabetes led to ROS accumulation in adipose tissue, senescence-like changes, and an increase in proinflammatory cytokine activities [33]. Interestingly, we observed the upregulation of p53 transcripts in ASC_EMS_ as well as in adipose tissue of EMS horses. It was found that the elevated expression of p53 in adipose tissue was significantly involved in the development of insulin resistance and promoted age-related changes [34]. Senescent cells play an important role not only in aging, but also in metabolic disorders [35]. Growth factors, cytokines, and hormones secreted by senescent adipocytes affect neighboring cells, including ASCs, thereby contributing to tissue dysfunction and chronic inflammatory state. This statement is supported by our previous study, where we have shown increased IL-6 and TNF*α* accumulation in adipose tissue of EMS horses [19].

Physiological impairment and ROS accumulation in ASC_EMS_ triggers a natural survival mechanism, autophagy, which allows maintaining cellular homeostasis. Autophagy is a dynamic process, switched on in response to stress and starvation leading to the removal of damaged organelles and misfolded proteins [36, 37]. Here, we observed an increased expression of LAMP2 mRNA in ASC_EMS_ undergoing adipogenic differentiation. Elevated LAMP2 expression indicates triggering a survival mechanism that protects against excessive oxidative stress in the course of adipogenic differentiation. The upregulation of LAMP2 transcription was observed not only in ASC_EMS_, but also in adipose tissue of EMS horses. It was demonstrated that LAMP2-deficient mice were protected against obesity, lipid accumulation, as well as hyperinsulinemia and hyperglycemia induced by a high-fat diet. Those results clearly indicated that the LAMP2-dependent formation of autophagosomes was involved in the adipogenic differentiation disorder [38]. A recent study has shown that autophagy is a common phenomenon in obese patients, as increased expression of Atg5, LC3A, and LC3B was observed in adipose tissue [22]. We hypothesize, that autophagy in adipose tissue of EMS horses can be triggered by insulin resistance, high levels of circulating glucose, and inflammation.

Furthermore, it is tempting to speculate, that selective mitophagy is a key mechanism that allows the removal of defective mitochondria and helps sustain proper cellular bioenergetics in ASC_EMS_ under adipogenic differentiation. As shown by Forni et al. [39], murine MSC differentiation is regulated by mitochondrial dynamics that involves mitochondrial fission/fusion processes. In turn, Mandal et al. [40] have shown that both proliferation and differentiation of ESCs are dependent on the proper function of mitochondria. In the current study, we observed elevated PARKIN expression in ASC_EMS_, which is selectively recruited to dysfunctional mitochondria with a low membrane potential. This clearly indicates that increased PARKIN expression promotes mitophagy in ASC_EMS_ and may be recognized as a protective mechanism that removes defective mitochondria from ASC_EMS_ in the course of adipogenic differentiation. The enhanced mitophagy in ASC_EMS_ is further supported by the observed elevated transcription of mitochondrial fission (Fis).

During adipogenic differentiation, ASC_EMS_ were characterized by increased methylation status and decreased expression of demethylation-related proteins, TET 2 and TET3. Progression from stem cells to differentiated cells requires certain changes in the gene expression profile, and epigenetic mechanisms are crucial in the process of differentiation [41]. We hypothesize that the observed hypermethylation in ASC_EMS_ is responsible for silencing genes involved in adipogenic differentiation, thereby affecting its effectiveness. However, further research must be carried out to confirm our presumption.

ER stress in adipose tissue is responsible for the development of inflammation and strongly affects the adipokine secretion profile. Recently, ob/ob diabetic mice have been shown to overexpress ER stress markers, such as BiP, phosphorylated PERK, and the phosphorylated *α*-subunit of eukaryotic translational initiating factor 2 (eIF2*α*) in adipose tissue [42]. Here, we observed elevated expression of CHOP and PERK mRNA in both ASCs and adipose tissue of EMS horses. ER stress activates the inflammasome, which leads to the secretion of proinflammatory cytokines that underlie the development of insulin resistance. This was consistent with our previous study, in which we showed that adipose tissue of EMS ponies was profusely infiltrated by macrophages and lymphocytes and contained high levels of IL-11, IL-6, and TNF-*α* [19]_._ In contrast, in the presented research, we found that IL-6 expression is decreased in ASC during adipogenic differentiation. Data regarding the role of IL-6 in MSC differentiation exists, however, in relation to osteogenesis. Research conducted by Huh and Lee [43] indicated that IL-6 promotes osteogenesis of ASC. On the other hand, Zhang et al. [34] showed that the anti-inflammatory properties of mouse ASCs in acute lung injury (ALI) are partially explained by the activation of IL-6 secretion. However, the role of IL-6 in the adipogenic differentiation of ASCs needs to be further elaborated.

The prevalence of obesity, metabolic syndrome, and diabetes has dramatically risen in recent decades. Excessive hypertrophy and hyperplasia of adipocytes are decisive determinants of abnormal adipose tissue growth, which contributes to the development of the abovementioned disorders. In the present study, we have shown that ASC_EMS_ are characterized by increased oxidative stress, senescence, and apoptotic phenotype. Moreover, for the first time we observed a high level of mitochondrial and ER stress caused by excessive ROS. Finally, we have discovered that triggering of selective mitophagy may serve as a rescue mechanism that allows ASC_EMS_ to maintain multipotency and survive in an unfavorable, proinflammatory microenvironment of adipose tissue. We thus, speculate that targeting mitophagy in ASC_EMS_ might become a novel therapeutic approach in the future, aimed at improving mitochondrial biogenesis and metabolism, thereby affecting cell fate.

## Figures and Tables

**Table 1:** Criteria for classifying the horses into the experimental and control groups.

Group	0 (number)	Sex	Main clinical parameters					
			Bw (kg)	BCS (1–9)	CNS (1–5)	Fasting insulin (mU/ml)	LEP (ng/ml)	CGIT : GLU in 45 min (mg/dl)
Healthy horse	1	f	610	6	1	7	3.21	74/p
	2	f	644	7	2	12	4.12	69/p
	3	f	627	7	2	9	2.87	71/p
	4	m	609	6	1	8	1.86	89/p
	5	m	649	7	2	14	3.56	80/p
	6	m	639	7	2	13	2.91	74/p

Mean ± SD			629.7 ± 15.7	6.5 ± 0.5	1.7 ± 0.5	10.5 ± 2.6	3.1 ± 0.7	76.2 ± 6.7

Horse with EMS	1	f	710	8	3	83	4.89	138/p
	2	f	726	9	3	67	5.19	141/p
	3	f	760	9	4	98	9.12	140/p
	4	m	709	8	3	73	8.49	136/p
	5	m	716	8	4	69	7.27	134/p
	6	m	746	9	4	82	8.36	146/p

Mean ± SD			727.8 ± 19.1	8.5 ± 0.5	3.5 ± 0.5	78.7 ± 10.5	7.2 ± 1.6	139.2 ± 3.8

Table 1 is reproduced from Basinska et al. [19] (under the Creative Commons Attribution License/https://www.ncbi.nlm.nih.gov/m/pubmed/25269712/), f: female, m: male, BW: body weight, BCS: body condition score, CNS: cresty neck score, CGIT: combined glucose-insulin test, SD: standard deviation, LEP: leptin, GLU: glucose, p: positive test results, and n: negative test results.

**Table 2:** Sequences of primers used in qPCR.

Gene	Primer	Sequence 5′–3′	Amplicon length (bp)	Accession no.
PPAR*γ*	F:	TCCCTGTTTGTGTACAGCCTT	191	XM_014846252.1
	R:	CTCCATGGCTGATTTCCCCT		

STAT5A	F:	AGATGCTGGCCGAGGTCAAC	212	XM_001494738.4
	R:	AGACTTGGCCTGCTGCTCAC		

SREBP-1c	F:	TCAGCGAGGCGGCTTTGGAGCAG	80	XM_008542859.1
	R:	CATGTCTTCGATGTCGGTCAG		

p53	F:	TTCCGCAAGAAGGAGGAACC	114	U37120.1
	R:	TTTGGACAGAACTGCACCCT		

p21	F:	GAAGAGAAACCCCCAGCTCC	241	XM_014853747.1
	R:	TGACTGCATCAAACCCCACA		

BCL-2	F:	TTCTTTGAGTTCGGTGGGGT	164	XM_014843802.1
	R:	GGGCCGTACAGTTCCACAA		

TET3	F:	CAGCCTGCATGGACTTCTGT	188	XM_014731089.1
	R:	GTTCTCCTCACTGCCGAACT		

TET2	F:	ATCCTGATCCTGGTGTGGGA	143	XM_005607932.2
	R:	CCTTGACAGGCACAGGTTCT		

DNMT1	F:	GGCGAAAGCGGACAATTCTG	90	XM_014741825.1
	R:	AGCGGTCTAGCAACTGGTTC		

BECLIN-1	F:	GATGCGTTATGCCCAGATGC	233	XM_014833759.1
	R:	AACGGCAGCTCCTCTGAAAT		

LC3	F:	TTACTGCTTTGCTCTGCCAC	213	XM_014835085.1
	R:	AGCTGCTTCTCCCCCTTGTA		

LAMP2	F:	GCACCCCTGGGAAGTTCTTA	147	XM_014831347.1
	R:	ATCCAGCGAACACTCTTGGG		

PINK	F:	GCACAATGAGCCAGGAGCTA	298	XM_014737247.1
	R:	GGGGTATTCACGCGAAGGTA		

PARKIN	F:	TCCCAGTGGAGGTCGATTCT	218	XM_014858374.1
	R:	CCCTCCAGGTGTGTTCGTTT		

MNF1	F:	AAGTGGCATTTTTCGGCAGG	217	XM_001495170.5
	R:	TCCATATGAAGGGCATGGGC		

FIS1	F:	GGTGCGAAGCAAGTACAACG	118	XM_001504462.4
	R:	GTTGCCCACAGCCAGATAGA		

EIF2*α*	F:	AGTCTTCAGGCATTGGCTCC	489	XM_001488848.5
	R:	CCGAGTGGGACATGTATCGG		

CHOP	F:	AGCCAAAATCAGAGCCGGAA	272	XM_001488999.3
	R:	GGGGTCAAGAGTGGTGAAGG		

PERK	F:	GTGACTGCAATGGACCAGGA	283	XM_014731045.1
	R:	TCACGTGCTCACGAGGATATT		

BIP	F:	CTGTAGCGTATGGTGCTGCT	122	XM_005606029.2
	R:	CATGACACCTCCCACGGTTT		

IL-6	F:	CGTCACTCCAGTTGCCTTCT	225	XM_014830631.1
	R:	GCCAGTACCTCCTTGCTGTT		

GLUT4	F:	TGGGCTCTCTCCGTGGCCATCTT	658	NM_001081866.2
	R:	GCTGCTGGCTGAGCTGCAGCA		

APLN	F:	TGGGCCTGAGATGGCTCTAT	819	XM_014729165.1
	R:	CCTCGGGGTTATTGTGGGAC		

GAPDH	F:	GATGCCCCAATGTTTGTGA	250	XM_014866500.1
	R:	AAGCAGGGATGATGTTCTGG		

Table 2 is reproduced from Kornicka et al. [10] (under the Creative Commons Attribution License/http://pubs.rsc.org/is/content/articlelanding/2016/ra/c6ra00029k), PPAR*γ*—peroxisome proliferator-activated receptor gamma, STAT5A—signal transducer and activator of transcription 5A, SREBP-1c—sterol regulatory element binding transcription factor 1 (SREBF1), p53—tumor protein P53 (TP53), p21—cyclin dependent kinase inhibitor 1A (CDKN1A), BCL-2—B-cell lymphoma 2, TET3—Tet methylcytosine dioxygenase 3, TET2—Tet methylcytosine dioxygenase 2, DNMT1—DNA methyltransferase 1, BECLIN-1, LC3—microtubule-associated protein 1 light chain 3 beta (MAP1LC3B), LAMP2—lysosomal-associated membrane protein 2, PINK1—PTEN induced putative kinase 1, PARKIN—Parkin RBR E3 ubiquitin protein ligase (PRKN), MNF1—mitochondrial nucleoid factor 1, FIS1—fission, mitochondrial 1, EIF2*α*—eukaryotic translation initiation factor 2A, CHOP—DNA damage inducible transcript 3 (DDIT3), PERK—eukaryotic translation initiation factor 2-alpha kinase 3 (EIF2AK3), BIP—heat shock protein family A (Hsp70) member 5 (HSPA5), IL-6—interleukin 6, GLUT4—solute carrier family 2 member 4 (SLC2A4), APLN—apelin, GAPDH—glyceraldehyde 3-phosphate dehydrogenase.
